# Crystal Structure of ChrR—A Quinone Reductase with the Capacity to Reduce Chromate

**DOI:** 10.1371/journal.pone.0036017

**Published:** 2012-04-27

**Authors:** Subramaniam Eswaramoorthy, Sébastien Poulain, Rainer Hienerwadel, Nicolas Bremond, Matthew D. Sylvester, Yian-Biao Zhang, Catherine Berthomieu, Daniel Van Der Lelie, A. Matin

**Affiliations:** 1 Department of Biology, Brookhaven National Laboratory, Upton, New York, United States of America; 2 Department of Microbiology and Immunology, Stanford University, Stanford, California, United States of America; 3 CEA, DSV IBEB, Laboratoire des Interactions Protéine-Métal, Saint-Paul-lez-Durance, France; 4 CNRS, UMR Biologie Végétale et Microbiologie Environnementale, Saint-Paul-lez-Durance, France; 5 Université d'Aix-Marseille, Saint-Paul-lez-Durance, France; 6 Université d'Aix-Marseille, Laboratoire de Génétique et de Biophysique des Plantes, Marseille, France; 7 CEA, DSV IBEB, Marseille, France; 8 Discovery and Analytical Sciences, RTI International, Research Triangle Park, North Carolina, United States of America; University of Minho, Portugal

## Abstract

The *Escherichia coli* ChrR enzyme is an obligatory two-electron quinone reductase that has many applications, such as in chromate bioremediation. Its crystal structure, solved at 2.2 Å resolution, shows that it belongs to the flavodoxin superfamily in which flavin mononucleotide (FMN) is firmly anchored to the protein. ChrR crystallized as a tetramer, and size exclusion chromatography showed that this is the oligomeric form that catalyzes chromate reduction. Within the tetramer, the dimers interact by a pair of two hydrogen bond networks, each involving Tyr128 and Glu146 of one dimer and Arg125 and Tyr85 of the other; the latter extends to one of the redox FMN cofactors. Changes in each of these amino acids enhanced chromate reductase activity of the enzyme, showing that this network is centrally involved in chromate reduction.

## Introduction

Chromate [Cr(VI)] and other soluble forms of heavy metals and radionuclides are serious environmental pollutants in many hazardous environments, including the U.S. Department of Energy waste sites and corresponding facilities worldwide [1]. The use of *Shewanella* and *Geobacter* bacteria has been intensely studied as a potential cost effective solution to this problem. These bacteria can convert the soluble form of many such contaminants, e.g., Cr(VI) and uranyl [U(VI)], to their insoluble valence state of Cr(III) and U(IV), thus making it possible to prevent their spread. While unique features of the membrane-bound electron transport chain enable these bacteria to mediate the dissimilatory reductions [2], [3], we showed that nearly all bacteria possess an additional means of chromate reduction, involving soluble enzymes [4], [5], [6]. However, the effectiveness of bacteria utilizing either mechanism to reduce chromate is hampered because of its toxicity [4], [5], [7], [8]. The latter arises mainly due to the fact that many bacterial metabolic enzymes, *e.g.*, glutathione reductase and lipoyl dehydrogenase, can vicariously reduce chromate and do so by one electron reduction [4], [5], [8]. This generates the highly reactive Cr(V) radical, which redox cycles. In this process, Cr(V) is oxidized back to Cr(VI), giving its electron to dioxygen and generating reactive oxygen species (ROS). Repetition of this process results in the generation of large quantities of ROS, which poison the cells due to oxidative stress and exhaustion of the cell's reducing power (NAD[P]H) [4], [9].

Conversion of Cr(VI) to Cr(III) involves the transfer of three electrons, and if it could be brought about in one step, it would obviate Cr(V) generation. We discovered the soluble ChrR class of enzymes in search for such a mechanism. This class of enzymes is widely distributed in bacteria [6], [10], and can greatly mitigate redox cycling not only by a one-step reduction of chromate to Cr(III) but also by preempting its reduction by cellular one-electron reducers [4], [5]. We have also shown that the *Escherichia coli* ChrR enzyme can bring about ‘safe’ reduction of uranyl as well (by avoiding redox cycling) to its insoluble valence state of U(IV) [7]. Overproduction of ChrR in wild type *E. coli* cells markedly augmented their capacity to remediate chromate and uranyl [7] and since dissimilatory bacteria are also poisoned by heavy metals, the protective effect of such overproduction is likely to apply to them as well [4], [5], [9]. Reduction of metals/radionuclides by cellular soluble enzymes has the advantage that unlike the dissimilatory reduction, the reduced species is not directly released into the environment, where it might be subjected to re-oxidation [6]. Moreover, dissimilatory reduction is inhibited by nitrate and oxygen, which are commonly present in polluted environments; this is not the case with reductions mediated by the soluble enzymes [4], [7], [10], [11]. Another valuable feature of ChrR is that it can activate several cancer prodrugs and has proven useful in cancer chemotherapy, such as by our recently discovered prodrug 6-chloro-9-nitro-5-oxo-5H-benzo(a)-phenoxazine (CNOB) [12], [13].

The physiological role of the *E. coli* ChrR is quinone reductase, and this is likely to be the case for enzymes of this class also in other bacteria [14]. Reductions mediated by ChrR involve electron transfer from NADH to the various substrates via ping-pong bi-bi (double displacement) reaction resulting in the transfer of electrons from NADH to the enzyme bound flavin co-factor, which subsequently passes them on to the enzyme substrate [10]. No reactive radicals [like Cr(V), U(V), Q^•^ etc.] are thus generated, and redox cycling and ROS generation are eliminated or greatly minimized during reduction of diverse electrophiles [4], [5], [14].

In order to gain mechanistic insights into this important enzyme, which could afford rational means for its improvement, we report here the crystal structure of the *E. coli* ChrR enzyme at 2.2 Å resolution. The enzyme crystallizes as a tetramer, which is also its active oligomeric form. We also show that a hydrogen bond network involving four amino acids at the dimer-dimer interface and reaching towards the flavin mononucleotide (FMN) cofactor plays a central role in chromate reductase activity of ChrR.

## Materials and Methods

*Escherichia coli* BL21 (DE3) was cultivated in Luria-Bertani (LB) broth supplemented with kanamycin (30 µg/mL), or ampicillin (50 µg/mL). ChrR was generated and purified as described previously [4], [5], [9]. *E. coli* BL21 (DE3) and plasmid pET28a or pTYB3 (New England BioLabs, Ipwsich, MA) were used to generate histidine- or intein-tagged proteins, respectively. Protein expression was induced with isopropyl-ß-D-thiogalactopyranoside (IPTG, 0.1 mM) at 20°C for 20 h. The proteins were purified using His-Trap nickel, or chitin bead columns and the tags were removed either by thrombin cleavage kit (Novagen) or DTT treatment (IMPACT protein purification kit, New England BioLabs). Gelfiltration was performed for further purification of the proteins using a 26/600 Superdex 200 column (GE HealthCare, Piscataway, NJ) and 50 mM Tris-HCl buffer, pH 7.5, supplemented with 250 mM NaCl. For determining the protein oligomeric state, an analytic 10/300 Superdex 200 column was used, with an elution flow rate of 0.5 mL per min. The proteins were pre-incubated with FMN (1 mM, 2 h). They were quantified using Pierce BCA (Thermo Fischer Scientific, Waltham, MA) and Uptima (Interchim, Montluçon, France) kits, using bovine serum albumin as standard.

Site-directed mutagenesis was performed as described previously [7], [15], using appropriate primers (Table S1). These were designed to create single-codon mutations following the method of Kuipers et al [16]. The wild type *chrR* gene cloned into expression vector pET28a (Novagen, Emd Biochemicals, Gibbstown, NJ) was used as mutagenesis template. Verification that the desired mutations had been generated was obtained by sequencing. Proteins encoded by the modified genes were generated as described above. Chromate reductase activity was determined by measuring decrease in chromate concentration during enzyme assays, using the 1,5-diphenylcarbazide method as we have described previously [17]. The enzyme was assayed at 37°C in 0.3 mL reaction mixtures containing (final concentrations): Tris-HCl buffer (50 mM, pH 7), chromate (1 mM), NADH (4 mM) and 0.6 to 25 mg/L enzyme, depending on the samples. Two to three protein concentrations were used for each determination.

For crystal structure determination, the untagged wild type (WT) ChrR protein was concentrated to 26 mg/mL in Tris-HCl, NaCl (10 and 150 mM, respectively, pH 8.0), and crystallized by sitting drop vapor diffusion method, using PEG8000 (10%), sodium chloride (50 mM) and calcium acetate (100 mM) solution as precipitant. Hexagonal shaped crystals appeared after a day and grew to about 0.15×0.4×0.05 mm^3^ in three weeks. X-ray diffraction data of the native crystals were collected at a resolution of 2.2 Å at the X12C beamline of the National Synchrotron Light Source (Brookhaven National Laboratory, Upton, NY). Highly redundant data were collected by recording 360 images with 1° rotation of the crystal and processed using the HKL2000 suite [18]. The native crystals belonged to the hexagonal space group P6_3_22. The structure was solved by molecular replacement method (MR).

## Results

**Figure 1:**
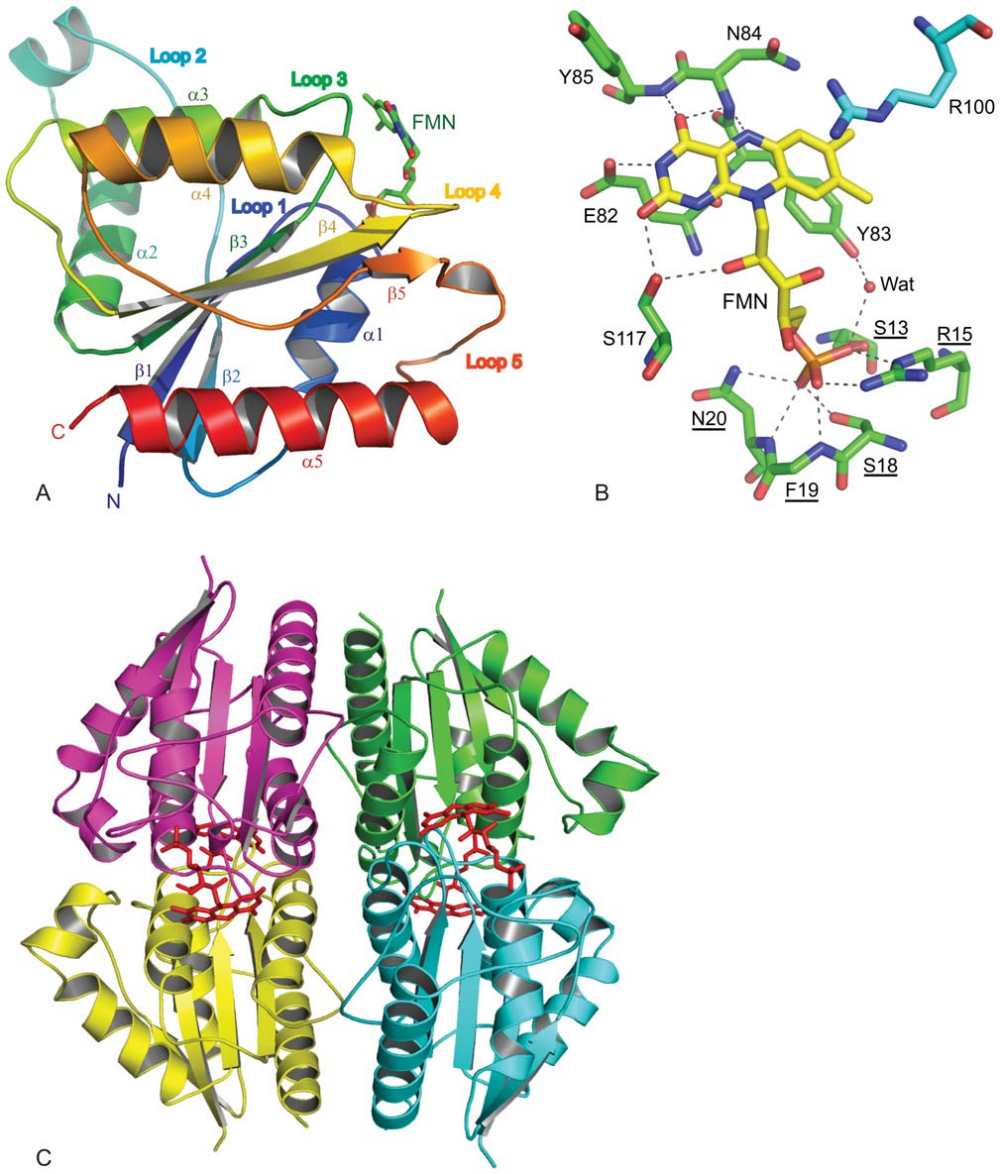
Crystal structure of ChrR. A: ChrR monomer; B: ChrR FMN-binding site; and C: ChrR tetramer. Underlined residues in panel B indicate NAD(P)H-dependent FMN-reductase signature sequence. Side chain atoms of F19 have been removed for clarity. The residue R100 belongs to the symmetry related molecule. Abbreviations: E = Glu; F = Phe; G = Gly; N = Asn; R = Arg; S = Ser; Y = Tyr; Wat = Water molecule.

The MR method used to solve the ChrR crystal structure employed the putative NADPH-dependent FMN-reductase T1501 of *Pseudomonas aeruginosa* PA01 [19] as model. Data collection details and refinement statistics are given in Table 1. The MR solution had a correlation coefficient of 0.302 between the two enzymes (R-factor 0.547) with two molecules in the asymmetric unit. This structure was modified with ChrR amino acid sequence and refined to a final R-factor of 0.404 (R-free 0.478). The phase values were calculated using this structure and a new model was built with *ARP_wARP* [20] through free atom calculations. The resulting model was checked and corrected manually using Program *O* [21] and refined with *CNS 1.1* [22]. This procedure eliminated any possible model bias and showed an initial R-factor of 0.302 (R-free 0.332). A difference electron-density map |Fo – Fc| (Fig. S1) calculated at 3.0σ using the completed protein structure showed an electron density compatible with FMN prosthetic group, which was included in the structure and further refined with water molecules (87 total). The same residual density was also observed in the composite omit map (not shown). Each monomer showed well-defined electron density for the protein and the FMN. The crystal structure (encompassing residues 4–184 in chain A and 4–185 in chain B) shows a flavodoxin fold (Fig. 1A), with a central β-sheet made up of five parallel β-strands in the order of β2, β1, β3, β4 and β5, and surrounded by helices α1 and α5 on one side, and α2 to α4 on the other. Loop 2 and loop 5 are long and more flexible than the other loops.

**Table 1:** Data collection and refinement statistics.

X-ray diffraction data		
Unit Cell	a:	107.25 Å
	b:	107.25 Å
	c:	128.25 Å
	α:	90.0°
	β:	90.0°
	γ:	120.0°
Space group:		P6_3_22
Resolution:		2.2 Å
Number of unique reflections:		22919
Completeness:		99.9%
R-merge^1^:		0.155
I/sig(I):		7.7
Redundancy:		24.5
Structure refinement		
R-work^2^:		0.240
R-free:		0.273
Number of reflections	R-work:	19964
	R-free:	807
Number of atoms refined	Protein:	2745
	Co-factor:	62
	Ion:	1
	Water molecules:	84
Overall B-value:		17.9 Å^2^
Root mean square deviations	Bond length:	0.007 Å
	Bond angle:	1.3°

1 R_sym_ = Σ_h_Σ_i_|I_i_(h)−<I(h)>|/Σ_h_Σ_i_ |I_i_(h)|, where I_i_(h) is the intensity measurement for a reflection h and <I(h)> is the mean intensity for this reflection.

2 R-value = Σ_i_∥F_i,obs_|−k|F_i,cal_∥/Σ_i_|F_i,obs_|.

FMN, representing the enzyme active site (Fig. 1A, B), is located in a shallow cavity surrounded by loops 1, 3 and 4 at the C-terminus of the β-sheet. FMN is anchored by several hydrogen bonds to a typical strand-loop-helix nucleotide-binding motif GSLRKGSFN (residues 12 to 20) located on loop 1 of the protein. The three FMN phosphate oxygen atoms are strongly attached to the protein. NH2 and NE groups of Arg15 form hydrogen bonds with phosphate atoms O1 and O3. These phosphate groups have additional interactions: O1 hydrogen bonds with N of Phe19, and O3 contacts OG of Ser13 directly, and OH of Tyr83 through a water molecule. Phosphate atom O2 binds within a 3.2 Å distance with OG of Ser18, and N and ND2 of Asn20. The flavin moiety also has multiple interactions: O2 hydrogen bonds with OG of Ser117; O4 with backbone nitrogens of Asn84 and Tyr85; and N3 with OE2 of Glu82. Moreover, Ser117 hydrogen bonds with O2 of ribitol, and the entire FMN molecule leans on loop 3 through van der Walls interactions.

The asymmetric unit is composed of two molecules forming a homo-dimer with a buried surface of 4670 Å^2^ (about 32%) for a total surface area of 14630 Å^2^. The hydrogen bonding interactions between the two monomers are listed in Table S2. In the dimer, the two FMN prosthetic groups locate at opposite sides of the interface with loop 2 from one monomer covering the prosthetic group of the other monomer. A potential metal binding site is observed in the dimer interface, involving residues Asn50 and Asp52 (loop 2) from one monomer and the carbonyl oxygen of Leu14 (loop 3) of the other. A calcium ion has been modeled at this site because the crystallization buffer contains calcium (Fig. S2).

**Figure 2:**
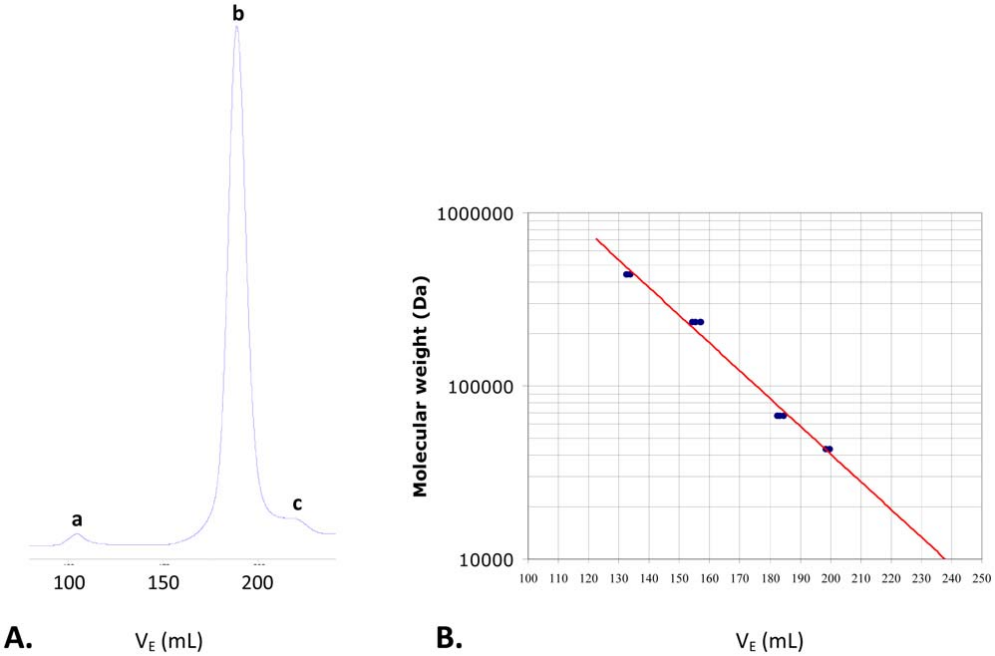
Oligomeric state of native ChrR and the mutants listed in Table 2 as determined by gel-filtration chromatography (Superdex 200 10/300). Panel A shows absorbance at 280 nm as a function of the elution volume, and Panel B is the calibration curve. The active enzymes are essentially tetrameric (peak *b*), as the dimer fraction (peak *c*) and protein aggregates (peak *a*) are negligible.

**Figure 3:**
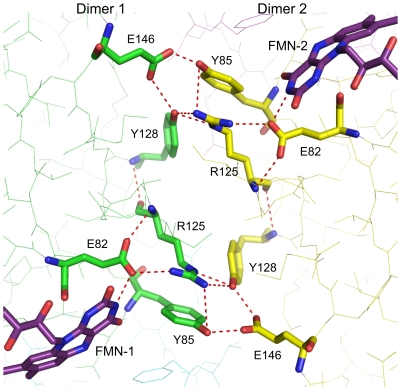
Hydrogen bonding network between FMN molecules from two different dimers of ChrR. Residue Tyr128 is closer to the FMN belonging to the symmetry-related dimer (8.4 Å) than to the FMN of the original molecule (14.1 Å). Abbreviations: E = Glu; R = Arg; Y = Tyr.

The oligomeric structure of the ChrR enzyme in the crystal is a tetramer, formed by two symmetry related dimers, as shown in Fig. 1C, with a buried surface area of 48% (12550 Å^2^, for a total tetramer surface area of 26050 Å^2^). It was possible that the tetramer seen in the crystals resulted from the high concentration of the protein used in making the crystals. However, size-exclusion chromatography at various protein concentrations, including those used for measuring the enzyme activity, showed that the active enzyme is indeed a tetramer (Fig. 2). Within the tetramer, the dimers interact by a pair of two hydrogen bond networks, each involving Tyr128 and Glu146 of one dimer and Arg125 and Tyr85 of the other with the latter extending to one of the redox FMN cofactors, as shown in Fig. 3. Bond distances are given in Table S3.

That Tyr128 is part of this network, and is closer to the FMN of the other dimer (8.4 Å) than the FMN of its own dimer (14.1 Å), was particularly notable to us because previous work from this lab has shown that substitution of this residue by Asn (mutant enzyme ChrR21) greatly improves chromate reductase activity of ChrR [7]. Size exclusion chromatography, as done with the wild type enzyme, showed that the active form of ChrR21 is also a tetramer (Fig. 2). We wondered what effect changes of other three individual amino acids in this network would have on chromate reductase activity. Given that Asn is less bulky than Tyr, we chose for these substitutions less bulky amino acids. Three additional mutants ChrR41 (Glu146Thr), ChrR42 (Tyr85Asn), and ChrR43 (Arg125Met) were thus generated by site directed mutagenesis. All were active as tetramers, as determined by the above method (Fig. 2), and all showed improved chromate reductase activity compared to the wild type enzyme (Table 2).

**Table 2:** Chromate reductase activity of ChrR mutant enzymes.

Enzyme	nM Cr(VI) reduced/min/mg protein	Enhancement factor
ChrR WT	860 (±50)	
ChrR 21 - Tyr128Asn	18065 (±1123)*	×21
ChrR 41 - Glu146Thr	25014 (±1007)	×29
ChrR 42 - Tyr85Asn	11496 (±134)	×13
ChrR 43 - Arg125Met	8093 (±951)	×9,4

* ChrR21 activity has been previously reported [7]. It was re-determined since.

protein preparation procedure and reaction mixtures used in this study are different from those used previously.

## Discussion

The wild type ChrR crystallized as a tetramer; size exclusion chromatography under conditions used to measure chromate reductase activity revealed this oligomeric form for the active enzyme as well as the mutants listed in Table 2; and manipulation of the amino acid constituents at the dimer-dimer interface markedly improved the ChrR chromate reductase activity. Taken collectively, these findings point to a central role for the tetrameric configuration in chromate reductase activity of this enzyme. Tetrameric association may be essential for the activity of small flavoproteins like ChrR to accommodate their substrates.

**Figure 4:**
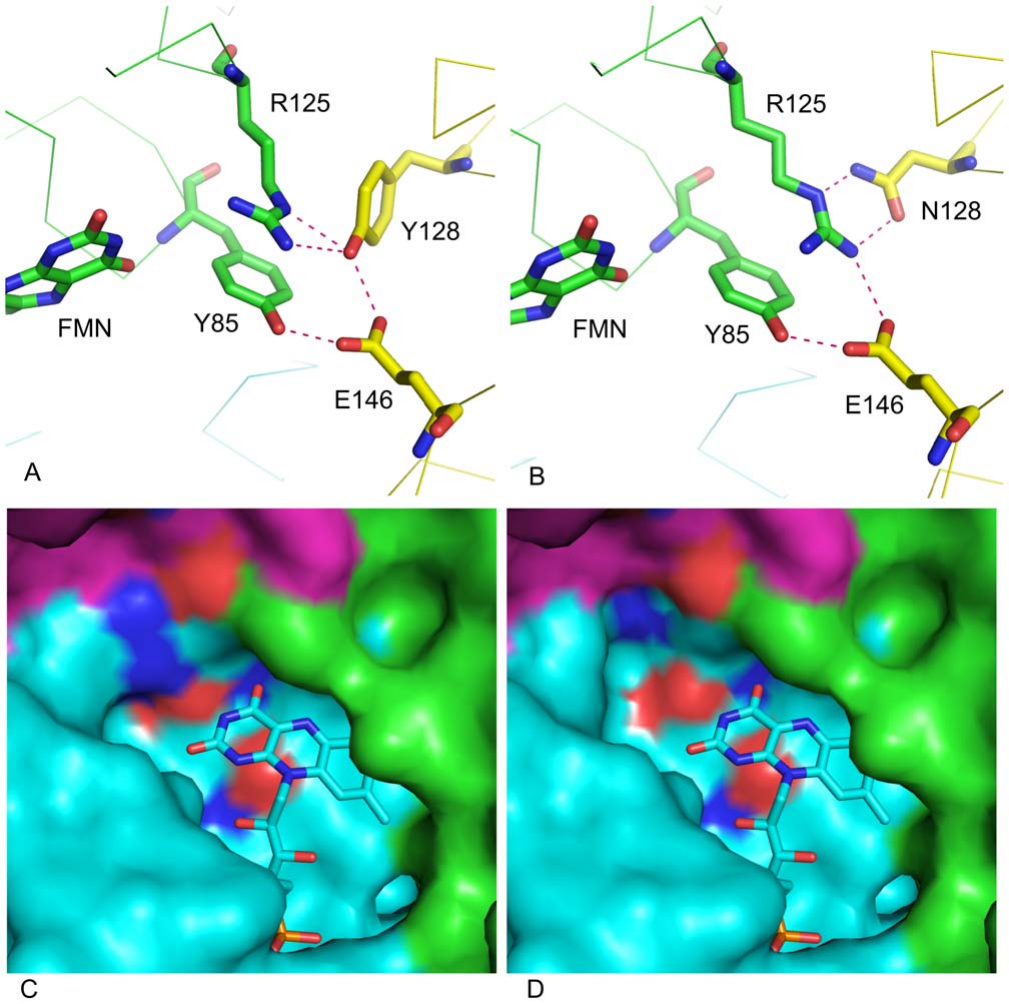
Diagramatic representation of the possible ways by which Tyr128Asn substitution in ChrR may increase chromate reductase activity. Fig. 4a and 4b show changes in hydrogen bonding between the dimers, and the surface diagrams (4c and 4d) show changes in the cavity around FMN. Both diagrams show the same location but for clarity the surface diagram view is rotated 90° on Z-axis and 45° on X-axis of the bonding diagram; Arg125 location is maintained at the top for direct comparison of the two views. See text for details.

Any definitive conclusions as to why the mutants shown in Table 2 were improved in chromate reductase activity must await co-crystallization of the wild type and the mutant enzymes with chromate and NADH. It is, however, useful for future studies to consider the potential mechanisms that may contribute to this improvement. The substitution of Tyr128 by Asn (ChrR21) is likely to have the following two effects. First, whereas in the wild type enzyme, three of the four hydrogen bonds between the dimers converge on a single oxygen atom of Tyr128 (Fig. 4a), Asn substitution would result in replacing them by individual bonds between separate appropriate donor and acceptor atoms [NE(R125)-H^…^ND2(Asn128), NH1(Arg125)-H^…^OD1(Asn128) and NH1(R125)-H^…^OE2(Glu146)] (Fig. 4b). Coupled with the fact that Asn is less bulky, the result is likely to be greater stability of the tetramer. A second potential effect concerns the cavity surrounding FMN. While in the native enzyme the Arg125 side chain reaches the solvent and restricts this cavity (Fig. 4c), the smaller size of Asn compared to Tyr would make the Arg side chain to turn inward to reach it, resulting in enlarged cavity surrounding FMN and enhancing FMN accessibility (Fig. 4d). Similar effects may contribute to the improved activity of the other mutant enzymes, in which also an original bulkier amino acid was replaced by a smaller one. Thus, in ChrR41, replacement of Glu146 by Thr would confer freedom of movement on the side chains of Tyr85 and Ty128, causing the side chain of Arg125 to move towards the other dimer, which would likely make the tetramer more stable and increase the accessible area of FMN. And in ChrR43, the smaller side chain of Met compared to Arg would enlarge the FMN cavity as in ChrR21 (Fig. 4d).

Additional factors are also likely to be involved. Tyr128 in the native enzyme is in indirect hydrogen bonding interaction with the FMN isoalloxazine N_3_H group, through Glu82 and Arg125 side chains in the wild type enzyme (Fig. 3). Its substitution by the shorter chain Asn may alter the hydrogen bonding interactions involving the side chain of Glu82, affecting in turn its interaction with ring III N_3_H group of FMN. The resulting change in the properties of FMN could therefore also have a role in modifying chromate reductase activity. Similarly, substitution of Arg125 by Met in ChrR43 can be expected to perturb Glu82 properties and hence properties of the FMN ring III.

Regardless, the results presented here indicate a critical role for the hydrogen bond network involving the four amino acids at the tetramer interface (Tyr128, Glu46, Arg125, Tyr85) in chromate reductase activity of ChrR. We have shown that ChrR21 retains its property for chromate reduction without Cr(V) formation [7]; it is likely that the other mutants of Table 2 also retain this character.

Structurally, ChrR belongs to the FMN reductase family of flavodoxin superfamily. Given its physiological role, it is part of the quinone reductase enzymes sharing the flavodoxin-like fold [23], [24]. This role is to catalyze quinone reduction by simultaneous two-electron transfer (avoiding formation of the reactive semiquinone intermediate), preempt reduction of diverse electrophiles (e.g., quinones and chromate) by the cellular one-electron reducers and generation of quinols that promote tolerance to ROS [9], [14]. It thus constitutes, like the *E. coli* WrbA protein, a bridge between bacterial and eukaryotic NAD(P)H:quinone oxidoreductases [23].

A search for protein data bases showed that ChrR bears the closest structural similarity to the putative FMN-reductase of *P. aeruginosa* PA01 T1501 (PDB ID: 1RTT) [19] (root mean square deviation, 1.1 Å for 174 atoms; 46% amino acid identity) – the protein used to solve ChrR structure, as noted. In the tetrameric form, T1501 also resembles ChrR in hydrogen bonding interactions at the dimer-dimer interface. Other tetrameric flavoproteins, such as ArsH or the quinone reductase YhdA, while bearing less than 25% sequence similarity with ChrR, also structurally resemble it (Z scores, 22.2 and 21.9, respectively). However, their intermolecular interactions are different. In ChrR, as stated, the dimer-dimer interaction involves a network of hydrogen bonds involving FMN, Glu82, Tyr85 and Arg125 of one dimer, and Tyr128 and Glu146 of the second dimer (Fig. 3). In contrast, the dimer-dimer interaction in YhdA and ArsH involves salt bridges between Lys109 and Asp137 [25], and extra loops from N- and C- terminal extensions, respectively [24], [26]. Whether these differences affect the substrate range of these enzymes remains to be determined.

One of our applied goals in the work on ChrR has been to generate engineered bacteria for superior bioremediation including their use at contaminated sites. Currently, there is reluctance to introducing engineered bacteria into the environment. As this may change, further improvement in bacterial capacity for *in situ* bioremediation by molecular engineering remains a worthwhile approach. In the meantime, however, the results reported here could already lead to improved *in situ* bioremediation for the following reason. We have seen that changes in four different amino acids individually caused a significant increase in chromate reductase activity and have shown that at least one of them (ChrR21) [7] retains the capacity for ‘safe’ chromate reduction, i.e., without Cr(V) generation. This suggests a strong possibility that bacteria containing such mutant enzymes may have naturally evolved in chromate contaminated sites to escape its toxicity. Isolation of such bacteria from the polluted sites and their use in bioaugmentation of these sites can thus be a promising approach to improve chromate bioremediation [27], [28], [29].

The atomic coordinates and structure factors (code 3SVL) have been deposited in the Protein Data Bank, Research Collaboratory for Structural Bioinformatics, Rutgers.

## Supporting Information

Figure S1 **The difference |Fo-Fc| map showing electron density compatible with flavin mononucleotide (FMN) prosthetic group.**
(DOCX)

Figure S2 **Structure of the ChrR dimer indicating the position of the calcium ion.**
(DOCX)

Table S1 **Primers used for site-directed mutagenesis.**
(DOCX)

Table S2 **Residues involved in dimer formation.**
(DOCX)

Table S3 **Distances of the hydrogen bonds (Å) presented in** **Figures 3** **and** **4** **.** Residue name followed by (S) represents the symmetry related partner involved in the hydrogen bond.
(DOCX)
