# Supplementary figures and images for: Crystal Structure of ChrR—A Quinone Reductase with the Capacity to Reduce Chromate
Source: PLoS One. 2012 Apr 27;7(4):e36017. doi: 10.1371/journal.pone.0036017 (PMC3338774; doi:10.1371/journal.pone.0036017)

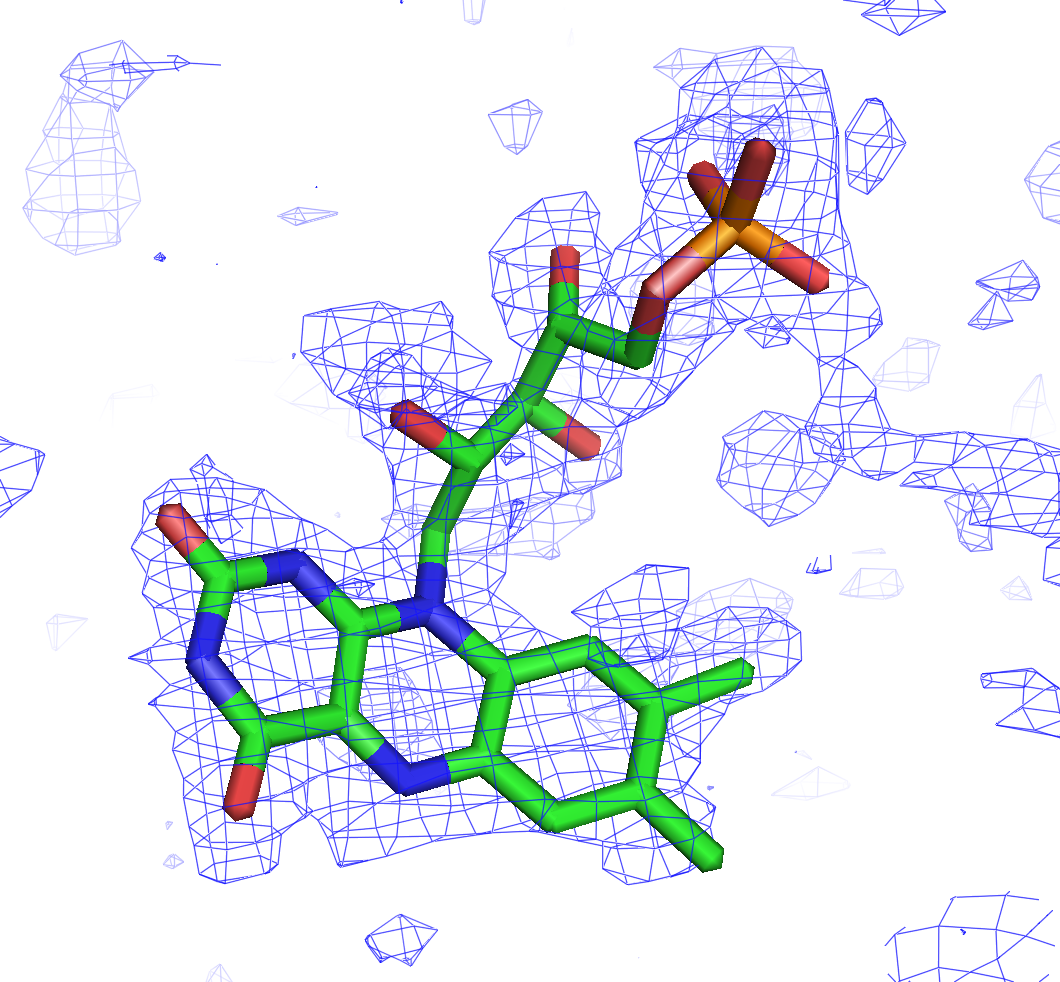

Supplement: Figure S1 — The difference |Fo-Fc| map showing electron density compatible with flavin mononucleotide (FMN) prosthetic group. (DOCX) [file pone.0036017.s001.docx]

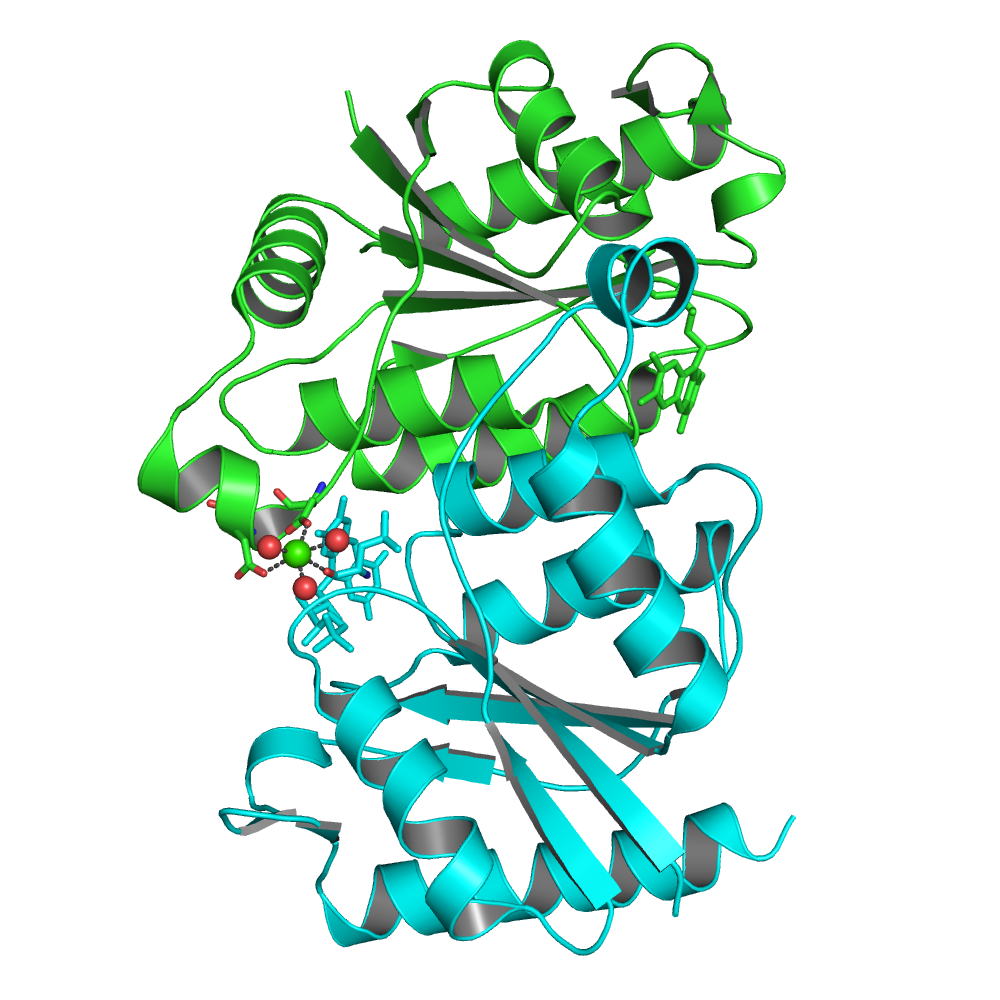

Supplement: Figure S2 — Structure of the ChrR dimer indicating the position of the calcium ion. (DOCX) [file pone.0036017.s002.docx]
