# Supplementary material for: Crystal Structure of ChrR—A Quinone Reductase with the Capacity to Reduce Chromate
Source: PLoS One. 2012 Apr 27;7(4):e36017. doi: 10.1371/journal.pone.0036017 (PMC3338774; doi:10.1371/journal.pone.0036017)
Supplement: Table S1 — Primers used for site-directed mutagenesis. (DOCX) [file pone.0036017.s003.docx]

**Supplementary Table S1.** Primers used for site-directed mutagenesis

| **ChrR41** | Glu146Thr  *Forward* | 5’GGTGATGAACAAGCCGACCTTTATGGGCGGCGTG3’ |
| --- | --- | --- |
|  | *Reverse* | 5’-CACGCCGCCCATAAAGGTCGGCTTGTTCATCACC3’ |
| **ChrR42** | Tyr85Asn  *Forward* | 5’-CGCCGGAATATAACAACTCGGTACCGGGTG-3’ |
|  | *Reverse* | 5’-CACCCGGTACCGAGTTGTTATATTCCGGCG-3’ |
| **ChrR43** | Arg125Met *Forward* | 5’-GTGATTGGCGGCGCGACCTGTCAGTATCACCT-3’ |
|  | *Reverse* | 5’-AGGTGATACTGACAGGTCGCGCCGCCAATCAC-3’ |
