# Supplementary material for: Crystal Structure of ChrR—A Quinone Reductase with the Capacity to Reduce Chromate
Source: PLoS One. 2012 Apr 27;7(4):e36017. doi: 10.1371/journal.pone.0036017 (PMC3338774; doi:10.1371/journal.pone.0036017)
Supplement: Table S2 — Residues involved in dimer formation. (DOCX) [file pone.0036017.s004.docx]

**Supplemental Table S2. Residues involved in dimer formation**

**Dimer interface**

**Monomer A Monomer B Distance (Å)**

K16 N … OD2 D52 2.80

Y83 O … NZ K92 3.10

N84 OD1 … OD1 D96 3.09

N84 OD1 … OG S99 3.01

Y85 O … NE2 Q132 2.83

Y87 O … NZ K92 2.90

G89 N … OD2 D96 2.77

D52 OD2 … N K16 2.79

K92 NZ … O Y83 2.96

K92 NZ … O Y87 2.84

D96 OD1 … OD1 N84 3.16

D96 OD2 … N G89 2.88

S99 OG … OD1 N84 3.17

Q132 NE2 … O Y85 3.07
