# Supplementary material for: Crystal Structure of ChrR—A Quinone Reductase with the Capacity to Reduce Chromate
Source: PLoS One. 2012 Apr 27;7(4):e36017. doi: 10.1371/journal.pone.0036017 (PMC3338774; doi:10.1371/journal.pone.0036017)
Supplement: Table S3 — Distances of the hydrogen bonds (Å) presented in Figures 3 and 4 . Residue name followed by (S) represents the symmetry related partner involved in the hydrogen bond. (DOCX) [file pone.0036017.s005.docx]

**Supplemental Table S3. Distances of the hydrogen bonds (Å) presented in Figures 3 and 4. Residue name followed by (S) represents the symmetry related partner involved in the hydrogen bond.**

R125 O … N Y128 3.22

R125 N … OE1 E82 2.90

R125 NH1 … OE2 E82 2.93

R125 NE … OH Y128(S) 2.84

R125 NH2 … OH Y128(S) 3.11

R125 NH2 … OH Y85 3.53

E82 OE2 … N3 FMN 2.72

Y85 OH … OE1 E146(S) 2.57

Y128(S) OH … OE2 E146(S) 2.77
